# Supplementary material for: Zetaproteobacteria Pan-Genome Reveals Candidate Gene Cluster for Twisted Stalk Biosynthesis and Export
Source: Front Microbiol. 2021 Jun 18;12:679409. doi: 10.3389/fmicb.2021.679409 (PMC8250860; doi:10.3389/fmicb.2021.679409)
Supplement: Supplementary file 1 [file Data_Sheet_1.PDF]

Zetaproteobacteria pan-genome reveals candidate gene cluster for twisted stalk biosynthesis and export

Supplementary Information

Koeksoy, E., Bezuidt, O.M., Bayer, T., Chan, C.S., Emerson, D.

FIGURES

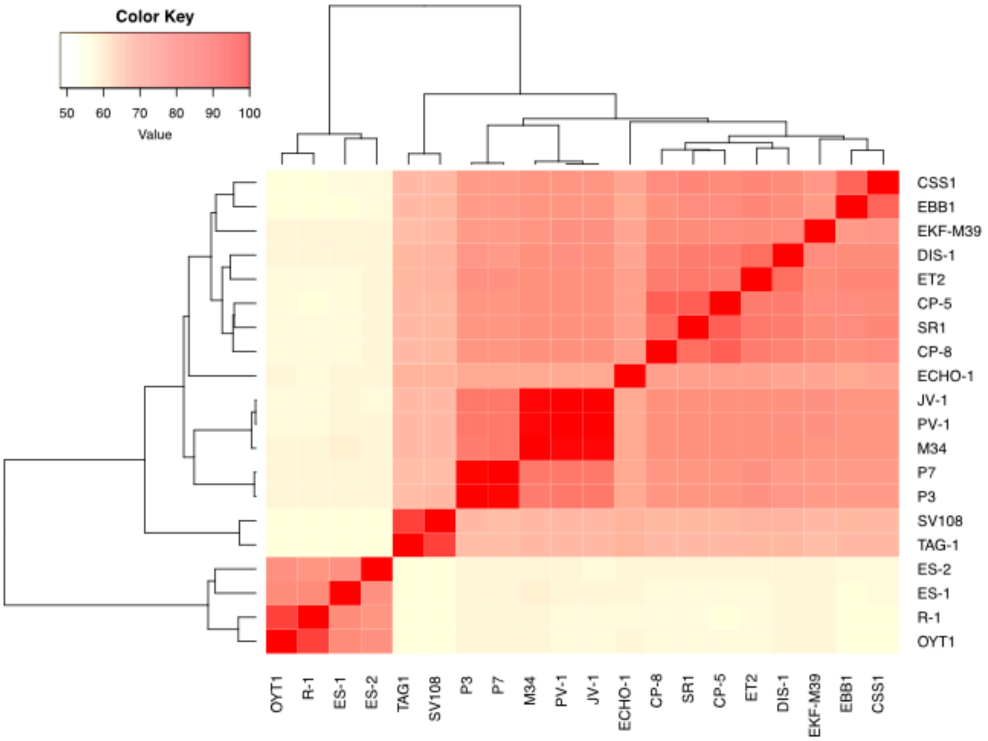

Figure S1: Degree of genomic similarity between 16 Zetaproteobacteria and 4 Betaproteobacteria genomes included in this study based on amino acid identities of their protein coding genes. Genomes belonging to the same species are represented in intense red (>95%), whereas lighter colors indicate genomes to fall below the species classification cutoff.

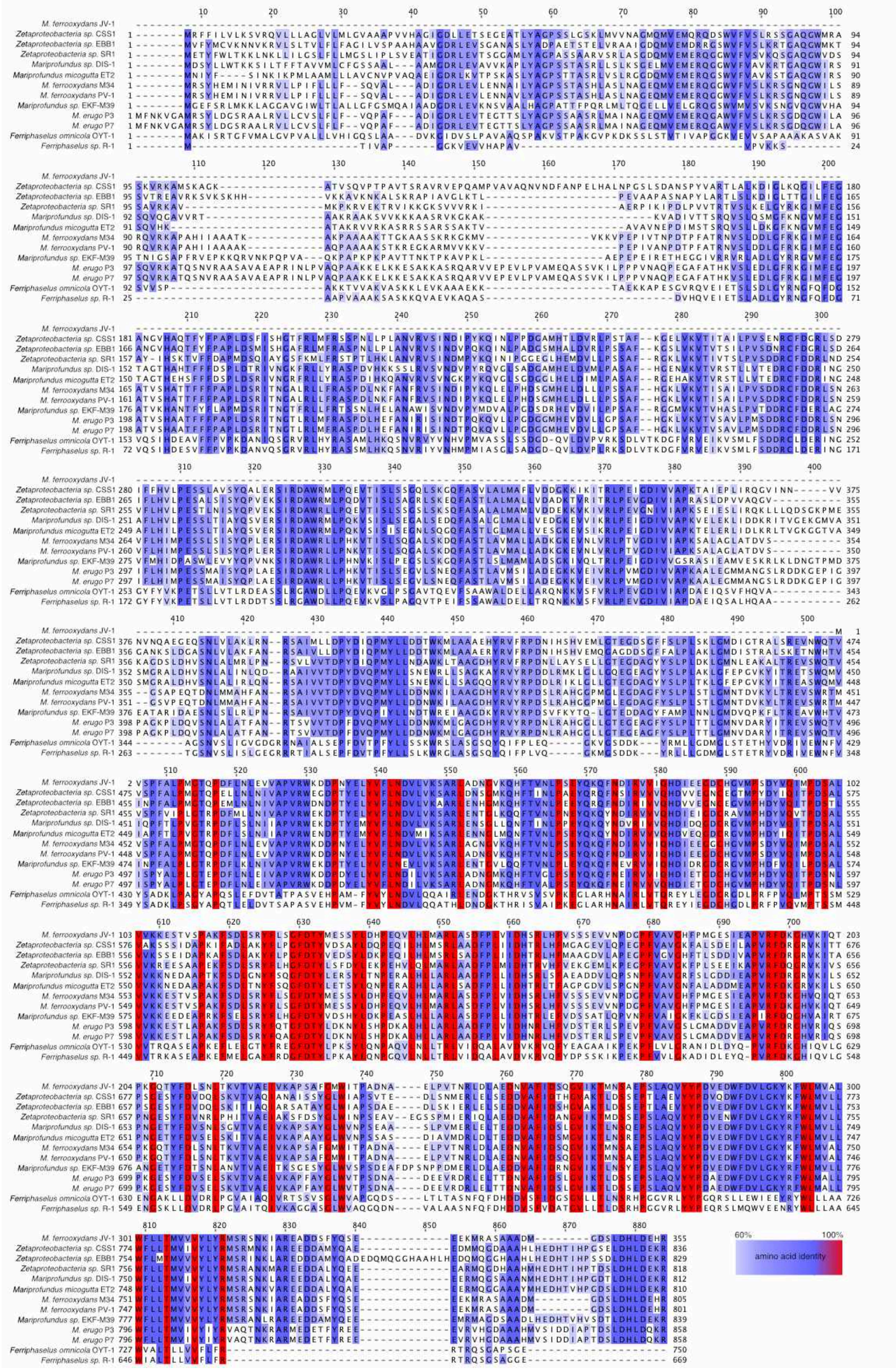
M. ferrooxydans JV-1
Zetaproteobacteria sp. CSS1
Zetaproteobacteria sp. EBB1
Zetaproteobacteria sp. SR1
Mariprofundus sp. DIS-1
Mariprofundus micogutta ET2
M. ferrooxydans M34
M. ferrooxydans PV-1
Mariprofundus sp. EKF-M39
M. erugo P3
M. erugo P7
Ferriphaselus omnicola OYT-1
Ferriphaselus sp. R-1
60%
100%
amino acid identity

Figure S2: Alignment of *sfz1 and sfb1* protein sequences in stalk-formers. 100% identical residues are highlighted in red, less conserved residues are shown above 60% amino acid identity.

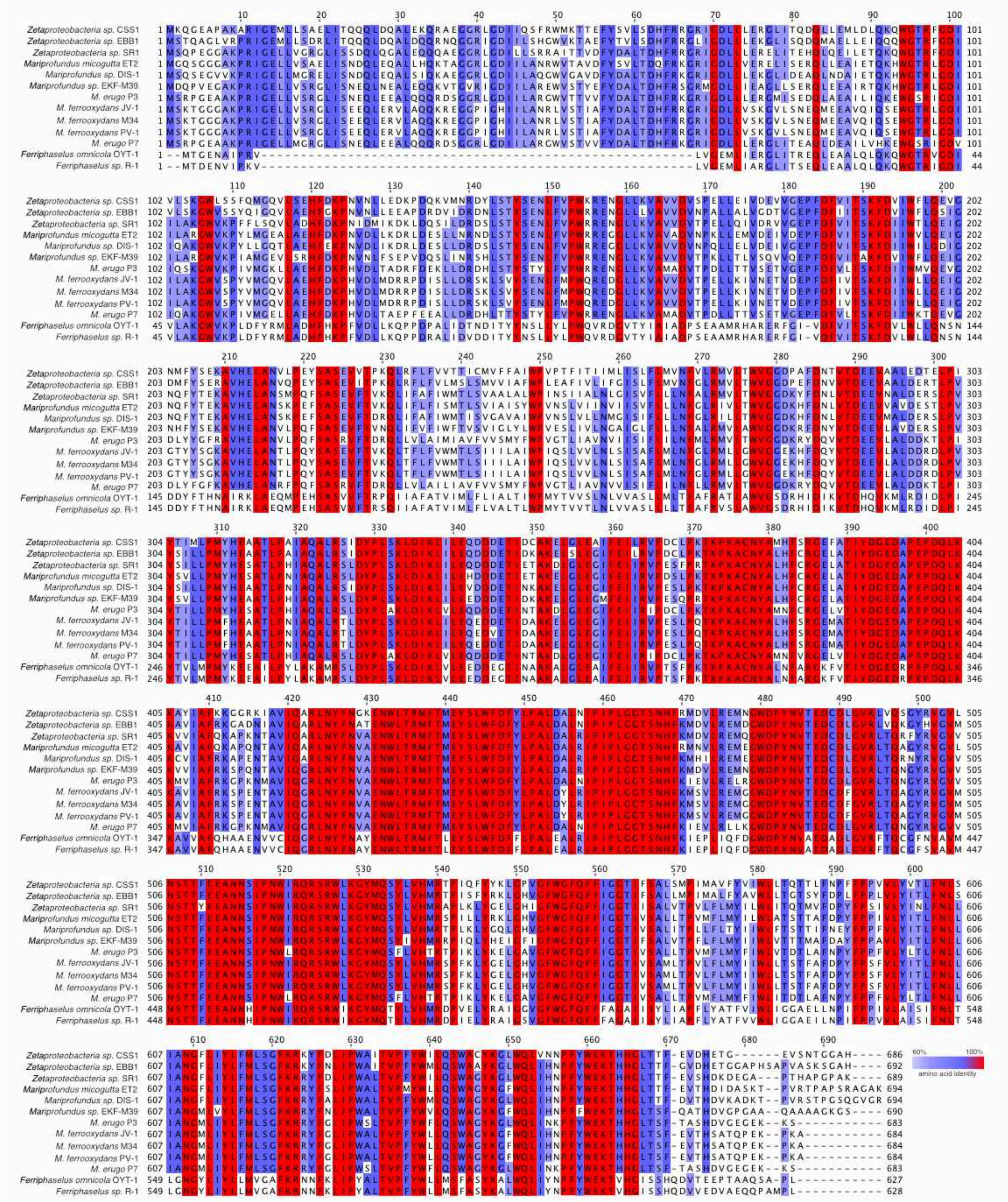

Figure S3: Alignment of *sfz2 and sfb2* protein sequences in stalk-formers. 100% identical residues are highlighted in red, less conserved residues are shown above 60% amino acid identity.

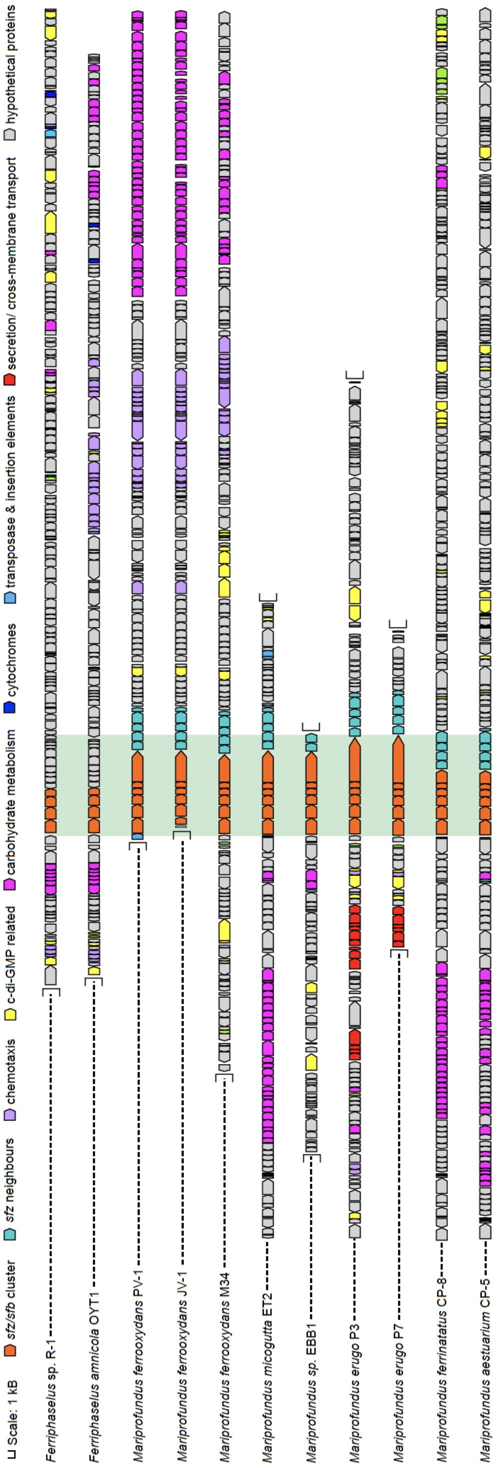

Figure S4: Gene synteny of the *sfz/sfb* cluster in stalk-forming Zetaproteobacteria and Betaproteobacteria. *Sfz* clusters are only shown for genomes that are already publicly available.

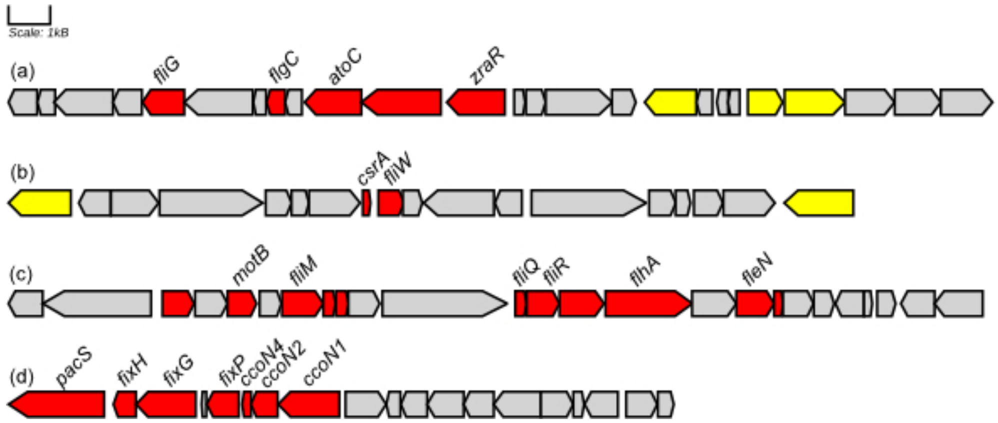

Fig. S5: Selected stalk-former unique genes in *M. ferrooxydans* PV-1 (red) with genes that are not unique to stalk-formers (grey) and genes with a potential function in c-di-GMP signaling (highlighted in yellow).

Supplementary Information - TABLES

Table S1: Relative proportions of core, accessory, and strain-specific genes in Zetaproteobacteria pan-genomes computed between 30-90% minimal blastp identity.

	Minimal blastp identity						
	30%	40%	50%	60%	70%	80%	90%
**Core**	10.2	10.3	8.5	5.4	2.4	0.7	0.04
**Accessory**	48.0	46.5	44.4	42.8	38.9	31.4	22.2
**Strain-specific**	41.8	43.2	47.1	51.8	58.7	67.9	77.7

Table S2: Proportions of core, strain-specific, and accessory genes in each isolate genome extracted from the Zetaproteobacteria pan-genome computed at 50% min. blastp identity. Shown values are percentages of total gene numbers.

Genome	Source environment	Core	Accessory	Strain-specific
M. ferrooxydans JV-1	deep sea	30.8	66.7	2.5
M. ferrooxydans PV-1	deep sea	30.6	66.3	3.1
M. ferrinatatus CP8	estuarine bay	38.0	55.3	6.7
M. erugo P3	steel coupon	32.6	60.5	7.0
M. ferrooxydans M34	deep sea	32.3	60.2	7.4
Zetaproteobacteria sp. SR1	worm burrow	36.3	54.6	9.1
M. erugo P7	steel coupon	31.5	59.1	9.3
M. aestuarium CP5	estuarine bay	35.1	54.4	10.4
M. micogutta ET2	coastal bay	35.2	54.0	10.8
G. bivora TAG-1	deep sea	39.0	47.4	13.6
G. bivora SV-108	deep sea	38.5	45.0	16.5
Zetaproteobacteria sp. EBB1	FeS coupon	31.9	51.4	16.7
Mariprofundus sp. EKF-M39	deep sea	32.0	51.0	16.9
Mariprofundus sp. DIS-1	coastal bay	29.6	52.7	17.6
Zetaproteobacteria sp. ECHO1	worm burrow	44.2	36.3	19.4
Zetaproteobacteria sp. CSS1	worm burrow	31.5	48.3	20.2

Table S3: Stalk-former unique genes extracted from Zetaproteobacteria pan-genomes computed at minimal blastp identities between 30-80%. Rows representing *sfz1-6* are shaded in light grey. Genes with potential functional links to a switching response between sessile and motile growth are marked with *.

Min. blastp identity [%]	Gene	Annotation (prokka)	Min. length [bp]	Max. length [bp]	Average length [bp]
80	*ccoN1*	Cbb3-type cytochrome c oxidase subunit CcoN1	1418	1466	1439
	*flgC**	Flagellar basal-body rod protein FlgC	446	449	446
	*flgG**	Flagellar basal-body rod protein FlgG	551	788	765
	*flhA**	Flagellar biosynthesis protein FlhA	2081	2087	2084
	*fliG**	Flagellar motor switch protein FliG	1001	1019	1007
	*fliN**	Flagellar motor switch protein FliN	296	320	302
	*fliQ**	hypothetical protein	224	353	272
	*csrA**	Carbon storage regulator	254	272	259
	HP_80_1	hypothetical protein	149	206	174
	HP_80_2	hypothetical protein	641	710	653
	HP_80_3	hypothetical protein	746	755	749
	*pomA**	Chemotaxis protein PomA	758	758	758
	Ppa	Inorganic pyrophosphatase	599	635	618
	*zraR**	Transcriptional regulatory protein ZraR	1388	1397	1392
70	*atoC**	Regulatory protein AtoC	1355	1367	1361
	*cheB**	Chemotaxis response regulator protein-glutamate methylesterase	2189	2261	2208
	*flgE**	Flagellar hook protein FlgE	1361	1730	1482
	*flgH**	Flagellar L-ring protein	695	701	695
	*flhB_1**	Flagellar biosynthetic protein FlhB	1067	1070	1067
	*flhB_2**	Flagellar biosynthetic protein FlhB	275	308	286
	*fliF**	hypothetical protein	1637	1658	1645
	*fliI**	Flagellum-specific ATP synthase	1361	1454	1405
	*fliR**	hypothetical protein	779	782	781
	**HP_70_1**	**hypothetical protein (*sfz2*)**	**2051**	**2084**	**2064**
	HP_70_2	hypothetical protein	398	410	400
	HP_70_3	hypothetical protein	1577	1619	1589
	HP_70_4	hypothetical protein	224	323	300
	HP_70_5	hypothetical protein	566	602	575
	motB	Motility protein B	698	701	698
	ylxH	Iron-sulfur cluster carrier protein	857	956	901
60	*fixP*	Cbb3-type cytochrome c oxidase subunit FixP	851	869	862
	*flgB**	Flagellar basal body rod protein FlgB	401	407	402
	*flgG**	Flagellar basal-body rod protein FlgG	740	743	742
	*flgK**	Flagellar hook-associated protein 1	1727	1745	1731
	*fliM**	Flagellar motor switch protein FliM	986	992	989
	*fliW**	Flagellar assembly factor FliW	407	458	438
	**HP_60_1**	**hypothetical protein (*sfz1*)**	**1067**	**2576**	**2353**
	HP_60_2	hypothetical protein	842	1097	924
	HP_60_3	hypothetical protein	569	596	579
	HP_60_4	hypothetical protein	1400	1427	1414
	HP_60_5	hypothetical protein	212	248	220
	**HP_60_6**	**hypothetical protein (*sfz3*)**	**1499**	**1535**	**1509**

	HP_60_7	hypothetical protein	482	497	485
	HP_60_8	hypothetical protein	626	644	636
	HP_60_9	hypothetical protein	1277	1304	1285
	HP_60_10	hypothetical protein	380	407	389
	HP_60_11	hypothetical protein	476	551	505
	pal	hypothetical protein	620	758	656
	rcsC	Sensor histidine kinase RcsC	1079	2114	2007
50	*flgD**	Basal-body rod modification protein FlgD	665	683	671
	**HP_50_1**	**hypothetical protein (*sfz6*)**	**3311**	**7352**	**4899**
	HP_50_2	hypothetical protein	695	737	713
	HP_50_3	hypothetical protein	686	785	720
	HP_50_4	hypothetical protein	278	344	318
	HP_50_5	hypothetical protein	1520	1541	1530
	HP_50_6	hypothetical protein	1907	1985	1952
	HP_50_7	hypothetical protein	254	263	257
	HP_50_8	hypothetical protein	380	404	383
	HP_50_9	hypothetical protein	641	674	655
	HP_50_10	hypothetical protein	212	230	219
	HP_50_11	hypothetical protein	440	518	482
	**HP_50_12**	**hypothetical protein (*sfz4*)**	**971**	**1037**	**1006**
	**HP_50_13**	**hypothetical protein (*sfz5*)**	**929**	**983**	**946**
	HP_50_14	hypothetical protein	281	350	301
	HP_50_15	hypothetical protein	1406	1439	1430
	pacS	putative copper-transporting ATPase PacS	2291	2591	2423
	pckA	Phosphoenolpyruvate carboxykinase (ATP)	995	1595	1532
40	HP_40_1	hypothetical protein	701	1007	759
	HP_40_2	hypothetical protein	437	452	444
	HP_40_3	hypothetical protein	458	494	464
	HP_40_4	hypothetical protein	1619	2213	1763
	HP_40_5	hypothetical protein	497	509	500
	HP_40_6	hypothetical protein	1436	1472	1444
	HP_40_7	hypothetical protein	293	398	318
	HP_40_8	hypothetical protein	623	809	716
	HP_40_9	hypothetical protein	1010	1526	1238
30	HP_30_1	hypothetical protein	809	818	812
	HP_30_2	hypothetical protein	1229	1253	1239
	HP_30_3	hypothetical protein	1691	2018	1760
	HP_30_4	hypothetical protein	278	323	291
	HP_30_5	hypothetical protein	482	575	530
	rcsC	Sensor histidine kinase RcsC	401	2738	1940

Table S4: Top 10 COGs in strain-specific genes of the Zetaproteobacteria pan-genome computed at 50% minimal blastp identity.

COG category	COG functional annotation	Counts
COG3706	Response regulator containing a CheY-like receiver domain and a GGDEF domain	246
COG1196	Chromosome segregation ATPases	177
COG2197	Response regulator containing a CheY-like receiver domain and an HTH DNA-binding domain	175
COG2204	Response regulator containing CheY-like receiver, AAA-type ATPase, and DNA-binding domains	172
COG3437	Response regulator containing a CheY-like receiver domain and an HD-GYP domain	171
COG0784	FOG: CheY-like receiver	169
COG0745	Response regulators consisting of a CheY-like receiver domain and a winged-helix DNA-binding domain	166
COG4753	Response regulator containing CheY-like receiver domain and AraC-type DNA-binding domain	165
COG2202	FOG: PAS/PAC domain	156
COG4566	Response regulator	148

Amino acid sequences of stalk formation genes *sfz1-sfz6* in *M. ferrooxydans* PV-1

Sfz1$_{PV-1}$ (NCBI locus ID EAU54621)
MRSYHEMINIVRRVLLPIFLLLFSQVALADIGDRLEVLENNAVLYAGPSSTASHLASLNAGEQMVEMERQGG
WVFVSLKRSGNQGWILSRQVRKAPAHIIAAAAKAQPAAAAKSTKREGKARMVVKKVPEPIVANPDTPFATR
NVSLDDLGFRKGIMFEGATVSHATTFFFPAPLDSRITNGALRLLFRASPDLNKFANFRVSINDIPYKQLELPHDS
GMHELDLLLPSSAFHGKLVKVTISAILPVSDDRCFDDRLSNVFLHIMPESSLSISYQPLERSIRDAWRLLPNKVTI
SLSQGALSKDQFASTLAVMALLADKGKEVNLVRLPTVGDIVIAPKSALAGLATDVSGSVPEQTDNLMMAHF
ANRSAIVVTDPYDVQPMYLLDDNWKILAAGDHYRVYRPDSLRAHGGPMGLEGDAGFYSLPLSTLGMNTDV
KYLTREVSWRTMVSPFALPMGTQPDFLNLEVVAPVRWKDDPNYELYVFLNDVLVKSARLADNGVKQHFTV
NLPSEYQKQFNDIRVVIQHDIEEGDCHGVMPSDYVQIMPDSALVVKKESTVSPAKFSDLSRYFLSGFDTYMES
SYLDHPEQVLHLMARLASDFPLVIDHSRLHFVSSSEVVNPDGPFVAVGHFPMGESIEAPVRFDKGHVKIQTPK
GQTYFDLSNLTKVTVAEIVKAPSAFGMWITPADNAELPVTNRLDLAEDNVAFIDSQGVIKTMNSAEPSLAQV
YYPDVEDWFDVLGKYKFWLMVALWFLLTMVVVYLYRMSRSNKIAREADDSFYQSEEEKMRASAAADMGD
SLDHLDEHR

Sfz2$_{PV-1}$ (NCBI locus ID EAU54620)
MSKTGGGAKPRIGELLVSRGLISEEQLERVLAQQKREGGPIGHIILANRLVSTIAFYDALTDHFRRGRIGDLLVS
KGVLSNEQMEEAVQIQSEWGTRLGDIILAKGWVSPYVMGQVLAEHFDKPHVDLMDRRPDISLLDRSKLSVY
SENLFMPWQREDGLLKVAVVDVTPELLKIVNETVDEPFDFIVTSKFDIIWLLQEIGGTYYSGKAVHELANTLPQ
YSASEVFTVKQLTFLFVWMTLSIIILAIWPIQSLVVLNLSISAFLMLNFGLRMLLGWVGGEKHFDQYVTDEEVL
ALDDRDLPVYTILLPMFHEAATLPNIAQALRTLDYPLSKLDIKLILEQEDDETIDAAKELGLEGIFEIIRVPESLPQT
KPKACNYALHFSRGEMATIYDGEDAPEPDQLKKAVIAFRKSPENTAVIQGRLNYFNVAENWLTRMFTMEYSL
WFDFYLPALDYLRIPIPLGGTSNHFKMSVLREMGGWDPYNVTEDCDFGVRLTQAGYRVGVMNSTTFEEAN
NSIPNWIRQRSRWLKGYMQSYLVHMRSPFKLYGELGHVGFWGFQFFIGGTIVSAMLTPVLFLMYIIWLLTST
FAFDPYFPSFVLYITLFNLLIANGMLIYLFMLSGFKRRYFGLIPWALTVPFYWLLQSWAGYKGFWQLIHNPFY
WEKTHHGLTSFEVTHSATQPEKPKA

Sfz3$_{PV-1}$ (NCBI locus ID EAU54619)
MNISPVRQRNYGGKAVLALIALAMCGVTIWLLSNDFQLAAATQRWAMVTGALGASDVLLENLSFLQPHFPL
YVLVPFYFIPGLDTGAAPYLVALLAATFLLFMWDRHLKEVEISGFRHALLAILIVSHPAFLWAATSGGHLMLSM
IAFYMLYRSAQHVITEHDIHSYISLAVVFLIFFFVDSSAIFIFVALLPLLVVIAPIRTVMVSPVGLYLIVGTPFAFAV
GTWAYMNWIFEGDPLFFITNADSAFMGGMLHIQDFPWLQQYGGQFFMPLIAATGYMLVAYPVSVYLLLD
TMDNSYRFRASFVLLLHPLIAIAIATSQYYLMHPFEILGLLSAGVMAELTFIKMQSRREFVLLVIFMFISSVGGW
WLFSQAGNPEMKHWMQALQGDTHQDSSNDADLALGLWLKHNRQQTMLYDRDAYEVIAARGDAKGLVL
SFSNEYKSNIRERIPNVAQIAVPDPTTVRGRRDKLNIRYPNLYDFGMKGFSIVYDHLGWRVYRKKHV

Sfz4$_{PV-1}$ (NCBI locus ID EAU54618)
MSDRRYFPVALALALLMTGCASHGLLQNSSMKHGFNVMQHASMSWEDPQARRSLREMVSTGANAVVLIS
FLKQPRPDSVEVTRSDAVSVNELKKAIRYARELGLYTVLKPQILVQGSWAGDVDPGRPQAWHQWFENYSRE
IVAFARFASEQRVQALVIGTELVHARDQVDWPELIRKVRAVYSGTITYAAHNVEGVKKFPYWNMLDTVSLTL
YPSLGSSGSRDDMQLYVDQAVENLHQAVSAYNKPLWVLEFGMPSARGASARPWAWQGLRHAQVDFNV
QRDALDIWLRALDKPWVDGAFIWVWYSDYKPGRINDADYTPQNKPAERIIRRYWN

Sfz5$_{PV-1}$ (NCBI locus ID EAU54617)
MIFKAVCRPALMLSRVAGIITIFFALAPSVQAGVGMLDQGESLYNGSVNFSGGNKHWDVQRQVITSVCQRR
NVSLLQGYEYGYSYFHTIFASVNFAYRRCGQINRAARVNPITGRVRPGYTLIGGPNAGIGDVQVGVRTRLNHR
GTAAWEALLIIPTGYDNNNPSRLGRGALGLGLGLKFSSDVPNMLVKRSSWGWTLGTRYVYFFSGKGNVLTSF
LETQYAFTSTDFQQTGDYAGLRINHSVSFGRNGLQRQLFFNQVPRSMTNSDQITVQLRYSHAFTGTGWSTS
VHAGKTVFGRNNPIDYFAGMGLSYRWRD

Sfz6$_{PV-1}$ (NCBI locus ID EAU54616)
LINRNAILACALLWVALSAGSLSAAEGDTYALLAASSLRNGPDSAYAPVAALSAGDRVIEVARKGDWIKVRQA
SGSADGWLYAASVEQVSAKGGQTILAAGSAAASAVPTQTVAPVAITQSTPTIPPGGQNAQESKPAVPVVTE
PVVPNVIYQGKGQTITATDLLDAPQSQPIAAPAKQQSVSPSPAPAASHHPLAAESNTPAVDTTAVMPVQQD
QGKTLSPAQASPVVTPTRAADSPAGDQQASVPAEKTATTPIMLWDDAVFGQNQTGSSSQTAAASPSHDTA
AATDTASASTKPENVTPAPASDPTVARAADTTVYRFNRPSKLRAGPDSKYDIVGWGGVDSYADEIDHKGNW
IKIQMQVSKRTGWVYQPSLTPVKAKELPKQVAAPADPASPGAQVLVNETPASEKAAAKTTDQALEAGGKVD
SVQTAADEPAMKLLSDEHGQQVGLSDALSPSVPVDHTASDTVIEPAADKVSAPAAVETTATSDAAGTVYRF
NRPSKLRAGPDSKYDIVGWGGVDSYADEIDHKGNWVKIQMQVSKRIGWVYQPSLTLVKAAVLPKEPAVSV
TANSAEGSGAAASPTESTPVAATAAKTAVEPGPVMTAKPAPIAAAQPPVLTSGTKTAAKPESIKVAPQSEHAI
YRTTTIRKGPGSLSDIMGWAGAGAMVTVLAQQGGWVNVRMQESGRTGWIDIGSIQKEAPATVVAVKQKA
APAEAAVKQKSVAPAAAAESPAAEGKAAVPEVVALKSATPKAEAVPTASTATAPERNLYRFIRNSTLRAGPG
ANYDVVAWGGVDSYASELELKGDWIRVEMEVSKRIGWVYHSSLVLAKAGRNRSAPVTVAAEKVAKINPDQ
LYFFSQTSDLLAGPGRQFDRIGWVGRDESATIIDSKGDWRRVNMTISGKRGWVPADLLKLALATGEIIVDDA
KSTAPVKKTAIAAFSHYQQARVVKTATLRTVPSIDSGMVGWVAKGERVSVLEQKDGWMRVNPQQVGEKP
GWIRGSYLTLINAPPGVTMIGDGQPASAYSNLITHGKTFNYSHAALEQALYRIPIEEIELTIGEDDLKALFRKGIY
DQSAFPVDILQDKRKLTGTIQVLGSSTRVFRKKSLRIKLDKDGGRWFGRRDIALRSMSSDKAMMREWMAW
KLMEAMGMKVPDVHFTRVRFNHGEKVALYLSVEWMGKEFFASNGLDPKGEFFQPNDAAHCGDLYTADN
MDICFDKITPQDGDYSSLSNMAKAMNAATSENMDQVLEEYFDDETVLNWIVANALVTNGDTYNKNYWLY
HQPGGGKWTMVPWDYNLTFGRTYDPYGVRPFTIFNDNFQYYYAPDVGAGNPLKDKALRNPVLRARIDAKI
KHLIGLEPNGPADTFGWFSPTVMEARIGNLASVIGKEVSKDTFLSYGKEDFTKTYESLMHYTKAHDYFLKYKLF
GRFDWQPDQPNPPLIDWPLPKELVGQGVIKAGTSQTHMVDTGWGYFVADLNLDKPLKKDTAFKVMVEG
GAAPRYLPPTKSASQCIQRSWVVSAETPGADVHGDVMFEYIQENSRRTEVPQTLHEDLLELWLLDGNRWKP
LKTDVNQYSNTLMARDIDMKYGEAKRFVACSPF
